# Identification and Analysis of the Acetylated Status of Poplar Proteins Reveals Analogous N-Terminal Protein Processing Mechanisms with Other Eukaryotes

**DOI:** 10.1371/journal.pone.0058681

**Published:** 2013-03-11

**Authors:** Chang-Cai Liu, Hang-Yong Zhu, Xiu-Mei Dong, De-Li Ning, Hong-Xia Wang, Wei-Hua Li, Chuan-Ping Yang, Bai-Chen Wang

**Affiliations:** 1 State Key Laboratory of Forest Genetics and Tree Breeding (Northeast Forestry University), Northeast Forestry University, Harbin, Heilongjiang, China; 2 Laboratory for Chemical Defense and Microscale Analysis, Hubei Nanxing General Chemical Factory, Zhijiang, Hubei, China; 3 Bureau of Garden and Park, Qitaihe, Heilongjiang, China; 4 Institute of Basic Medical Sciences, National Center for Biomedical Analysis, Beijing, China; 5 Key Laboratory of Photobiology, Institute of Botany, Chinese Academy of Sciences, Beijing, China; Institute of Botany, Chinese Academy of Sciences, China

## Abstract

**Background:**

The N-terminal protein processing mechanism (NPM) including N-terminal Met excision (NME) and N-terminal acetylation (N^α^-acetylation) represents a common protein co-translational process of some eukaryotes. However, this NPM occurred in woody plants yet remains unknown.

**Methodology/Principal Findings:**

To reveal the NPM in poplar, we investigated the N^α^-acetylation status of poplar proteins during dormancy by combining tandem mass spectrometry with TiO_2_ enrichment of acetylated peptides. We identified 58 N-terminally acetylated (N^α^-acetylated) proteins. Most proteins (47, >81%) are subjected to N^α^-acetylation following the N-terminal removal of Met, indicating that N^α^-acetylation and NME represent a common NPM of poplar proteins. Furthermore, we confirm that poplar shares the analogous NME and N^α^-acetylation (NPM) to other eukaryotes according to analysis of N-terminal features of these acetylated proteins combined with genome-wide identification of the involving methionine aminopeptidases (MAPs) and N-terminal acetyltransferase (Nat) enzymes in poplar. The N^α^-acetylated reactions and the involving enzymes of these poplar proteins are also identified based on those of yeast and human, as well as the subcellular location information of these poplar proteins.

**Conclusions/Significance:**

This study represents the first extensive investigation of N^α^-acetylation events in woody plants, the results of which will provide useful resources for future unraveling the regulatory mechanisms of N^α^-acetylation of proteins in poplar.

## Introduction

The N-terminal protein processing mechanism (NPM) represents a common protein modification that occurs in eukaryotes, and primarily involves the co-translational processes of N-terminal Met excision (NME) and N-terminal acetylation (N^α^-acetylation) [1]–[4]. In all eukaryotes, the nuclear-encoded protein synthesis machinery requires newly synthesized peptides to begin with methionine (Met), whereas plastid-encoded nascent proteins begin with a Met with an N-formyl group (Fo) [5]. Therefore, NME of the nuclear-encoded proteins requires only methionine aminopeptidase (MAP; EC 3.4.11.18) activity, which proteolytically removes the N-terminal Met [4], [6]. NME of the plastid-encoded proteins requires MAP activity and peptide deformylase (PDF) activity [7]. The latter enzymatic activity is required for the removal of the Fo groups, thereby unmasking the amino group of the first Met and allowing the subsequent action of MAP [1], [5], [8]. Following the synthesis of the peptides in eukaryotes, cytosolic MAPs may remove the first Met residue if the residue at position two has a small enough side-chain, resulting in N-terminal Ala, Val, Ser, Thr, Cys, Gly, or Pro amino acids [9]. Approximately two-thirds of mature proteins undergo NME induced by MAP [1]. Unlike eubacteria, which possess only one type of MAP (MAP1), eukaryotes possess a second type of MAP, MAP2, with similar substrate specificity as found for MAP1 [4]. Experimental data have shown that, in higher eukaryotes, MAP1s are found in mitochondria, plastids, and the cytoplasm, whereas MAP2s are found specifically in the cytoplasm, suggesting that NME occurs in all compartments where *de novo* protein synthesis occurs [1], [5], [10].

N^α^-acetylation is an enzyme-catalyzed reaction in which the protein α-amino group accepts an acetyl group from acetyl-CoA [9]. Currently, six types of Nats conserved from yeast to humans are responsible for these N^α^-acetylation events: each of the three major Nats, NatA, NatB and NatC contain a catalytic subunit, and one or two auxiliary subunits, whereas NatD, NatE and NatF are composed of only a catalytic subunit [11]–[12]. Each type of Nats appears to acetylate a distinct subset of substrates defined by the first N-terminal amino acid [13]. NatA is often responsible for the N^α^-acetylation of small N-terminal amino acid residues, including Ser, Ala, Thr, Val, Gly and Cys, following NME induced by MAP [2], [14]–[15]. Interestingly, NatF also has the potential to acetylate these types of N-termini where the Met has not been cleaved [12]. NatB potentially recognizes and acetylates Met-Asp-, Met-Glu-, and Met-Asn- N-termini [12]. Hydrophobic Met-Leu-, Met-Ile- and Met-Phe- are acetylated by NatC. Moreover, these hydrophobic termini are also recognized by NatF and NatE *in vitro*, suggesting that redundancy in activity also exists between particular Nats [12]. In yeast, NatD was found to acetylate the Ser-N-termini of histones 2A and 4 in vitro and in vivo, whereas no such activity has yet been observed in higher eukaryotes [16]. Furthermore, the entire genes encoding catalytic or auxiliary subunits of NatA-NatF have been identified and described in yeast and humans [12]–[13]. However, there is still no systematic and comprehensive characterization of Nats in *Arabidopsis* and poplar.

Previous evidence suggests that NPM possess similar mechanisms across several eukaryotes [1]–[2]. However, the NPM mechanism present in poplar remains poorly defined. Here, we identified 58 N^α^-acetylated proteins using tandem mass spectrometry combined with TiO_2_ enrichment of acetyl peptides in dormant terminal buds of poplar. The site-specific acetylation data provide a wealth of resources for decoding NPM mechanisms present in poplar. As far as we know, this study represents the first extensive investigation of N^α^-acetylation events in woody plants.

## Results

### Characterization of the Identified Acetylated Proteins in Poplar

The N^α^-acetylation of proteins was investigated to explore NPMs of woody plant proteins. Proteins from poplar were isolated and digested with trypsin in solution and the tryptic peptides were subjected to nanoUPLC-ESI-MS/MS for the identification of acetylation following TiO_2_ enrichment. The spectra representing all of these acetylated peptides and the original data collected are listed in the File S1. As outlined in the Table 1 and File S2, we have identified 58 N-terminally acetylated (N^α^-acetylated) proteins. These 58 proteins were divided into two groups: (i) the NME-independent N^α^-acetylation group, where the N-terminal Met residue (iMet) is retained and subsequently acetylated; and (ii) the NME-dependent N^α^-acetylation group, where the N-terminal iMet residue is removed and acetylation occurs at the exposed residue located at position two. In this study, most of N^α^-acetylated proteins (47, >81%) belong to group (ii), whereas the remaining proteins (11, ∼19%) belong to group (i), suggesting that N^α^-acetylation and NME could represent a common NPM of poplar proteins (Table 1 and File S2). Interestingly, we found that the N-terminus of sixteen identified N^α^-acetylated proteins (27.6%, 16/58) are also phosphorylated, which are respectively fifteen N^α^-acetylated proteins (31.9%, 15/47) of group (ii), and one N^α^-acetylated protein (9.1%, 1/11) of group (i) (File S2). Notably, phosphorylation of ten N^α^-acetylated proteins are present within the N-terminal regions while the N-terminal Ser residues of six proteins (37.5%, 6/16), including four translation initiation factor eIF-5A (717121, 832646, 835953 and 724093) and two metallopeptidase M24 proteins (819223 and 577003), were also found both N^α^-acetylated and phosphorylated (File S2). This similar event has also been observed in spinach chloroplasts, where N-terminal Ser residues of three proteins possess both phosphoryl and acetyl groups [17].

**Table 1:** Detailed information describing the N^α^-acetylation of poplar proteins.

NO.	JGI ID	Protein ID in NCBI	Description	Best hits in *Arabidosis*	E-value	Subcellular Location	Acetylated N-terminus	Putative Nats Type
Group (i): NME-independent N^α^-acetylation group								
1	644907	XP_002301543.1	S-adenosylmethionine synthetase 2	At4g01850	0.0	Cytoplasm	Ac-Met-Glu-Thr-	NatB
2	834837	XP_002320949.1	S-adenosylmethionine synthetase 2	At4g01850	0.0	Cytoplasm	Ac-Met-Glu-Thr-	NatB
3	729359	XP_002319463.1	S-adenosylmethionine synthetase 4	At3g17390	0.0	Cytoplasm	Ac-Met-Glu-Thr-	NatB
4	727847	XP_002316736.1	NAD(P)-binding Rossmann-fold-containing protein	At2g34460	9e^−137^	Chloroplast	Ac-Met-Glu-Ser-	NatB
5	711545	XP_002301809.1	Rotamase FKBP 1	At3g25230	0.0	Cytoplasm	Ac-Met-Glu-Glu-	NatB
6	742325	XP_002328616.1	Putative PR-10 type pathogenesis-related proteins	NA^a^		Cytoplasm	Ac-Met-Glu-Val-	NatB
7	552891	XP_002302922.1	1-aminocyclopropane-1-carboxylate oxidase-1	At1g06620	2e^−117^	Cytoplasm	Ac-Met-Glu-Val-	NatB
8	584641	XP_002331268.1	cAMP-regulated phosphoprotein 19-related protein	At5g64130	1e^−55^	Cytoplasm	Ac-Met-Glu-Asp-	NatB
9	657667	XP_002311836.1	Auxin/aluminum-responsive protein, putative	At5g19140	1e^−142^	Cytoplasm	Ac-Met-Leu-Gly-	NatC, or NatE, or NatF
10	814125	XP_002314449.1	HSBP (heat shock factor binding protein)	At4g15802	2e^−41^	Cytoplasm	Ac-Met-Asp-Gly-	NatB
11	794816	XP_002338396.1	Polyphenol oxidase	NA		Cytoplasm	Ac-Met-Gly-Asn-	NatF
Group (ii): NME-dependent N^α^-acetylation group								
12	557663	XP_002313984.1	Ran-binding protein 1-c	At5g58590	1e^−104^	Cytoplasm	Ac-Ala-Ser-Thr-	NatA
13	173568	XP_002298487.1	Ran-binding protein 1-b	At2g30060	7e^−105^	Cytoplasm	Ac-Ala-Ser-Thr-	NatA
14	568329	XP_002316672.1	Adenine nucleotide alpha hydrolases-like protein	At1g11360	5e^−108^	Cytoplasm	Ac-Ala-Ser-Ser-	NatA
15	815719	XP_002298802.1	L-galactose dehydrogenase	At4g33670	0.0	Cytoplasm	Ac-Ala-Ser-Pro-	NatA
16	836295	XP_002326641.1	Peroxin 19-1	At3g03490	3e^−110^	Cytoplasm	Ac-Ala-Asp-Gln-	NatA
17	817164	XP_002304276.1	Peroxin 19-1	At3g03490	2e^−116^	Cytoplasm	Ac-Ala-Asp-Gln-	NatA
18	561044	XP_002309211.1	NMT1 (protein N-myristoyltransferase 1)	At5g57020	0.0	Cytoplasm	Ac-Ala-Asp-Asn-	NatA
19	247052	XP_002320523.1	Nuclear transcription factor Y subunit B-3	At4g14540	1e^−75^	Cytoplasm	Ac-Ala-Asp-Ser-	NatA
20	832542	XP_002312089.1	Heat shock protein 70	At5g02500	0.0	Cytoplasm	Ac-Ala-Gly-Lys-	NatA
21	578767	XP_002325142.1	Glyceraldehyde-3-phosphate dehydrogenase	At2g24270	0.0	Cytoplasm	Ac-Ala-Gly-Lys-	NatA
22	563894	XP_002312090.1	Heat shock protein 70	At3g12580	0.0	Cytoplasm	Ac-Ala-Gly-Lys-	NatA
23	280896	XP_002332589.1	Heat shock protein 70	At3g12580	0.0	Cytoplasm	Ac-Ala-Gly-Lys-	NatA
24	675629	XP_002332588.1	Heat shock protein 70	At3g12580	0.0	Cytoplasm	Ac-Ala-Gly-Lys-	NatA
25	822482	XP_002316294.1	Heat shock protein 70	At3g12580	0.0	Cytoplasm	Ac-Ala-Gly-Thr-	NatA
26	817912	XP_002305213.1	HMGA (high mobility group protein A)	At1g14900	4e^−39^	Cytoplasm	Ac-Ala-Ala-Glu-	NatA
27	766915	XP_002312779.1	HMGA (high mobility group protein A)	At1g27090	7e^−129^	Cytoplasm	Ac-Ala-Ala-Thr-	NatA
28	724015	XP_002314993.1	S-adenosylmethionine synthetase 1	At1g02500	0.0	Cytoplasm	Ac-Ala-Glu-Thr-	NatA
29	564333	XP_002312296.1	S-adenosylmethionine synthetase 1	At3g17390	0.0	Cytoplasm	Ac-Ala-Glu-Thr-	NatA
30	589531	XP_002326399.1	DEAD-box ATP-dependent RNA helicase 38	At3g53110	0.0	Cytoplasm	Ac-Ala-Glu-Val-	NatA
31	203151	XP_002314085.1	RNA-binding protein 8A	At1g51510	4e^−86^	Cytoplasm	Ac-Ala-Asn-Thr-	NatA
32	172155	XP_002299789.1	RNA-binding protein 8A	At1g51510	4e^−89^	Cytoplasm	Ac-Ala-Asn-Thr-	NatA
33	648073	XP_002305183.1	Translation initiation factor eIF-5	At1g77840	0.0	Cytoplasm	Ac-Ala-Leu-Gln-	NatA
34	553039	XP_002302989.1	Translation initiation factor eIF-5	At1g36730	0.0	Cytoplasm	Ac-Ala-Leu-Gln-	NatA
35	590689	XP_002333033.1	Translation initiation factor eIF-5	At1g36730	0.0	Cytoplasm	Ac-Ala-Leu-Gln-	NatA
36	830730	XP_002303373.1	Plasminogen activator inhibitor 1	At4g17520	2e^−91^	Cytoplasm	Ac-Ala-Thr-Ala-	NatA
37	551211	XP_002302129.1	40S ribosomal protein S12-2	At2g32060	2e^−68^	Cytoplasm	Ac-Ser-Gly-Glu-	NatA
38	714910	XP_002306757.1	40S ribosomal protein S12-2	At2g32060	6e^−68^	Cytoplasm	Ac-Ser-Gly-Glu-	NatA
39	834953	XP_002321150.1	SUMO2 (small ubiquitin-related modifier 2)	At5g55160	8e^−58^	Cytoplasm	Ac-Ser-Gly-Val-	NatA
40	711526	XP_002301601.1	SUMO2 (small ubiquitin-related modifier 2)	At5g55160	4e^−58^	Cytoplasm	Ac-Ser-Gly-Ala-	NatA
41	717121	XP_002308400.1	Translation initiation factor eIF-5A	At1g13950	0.0	Cytoplasm	Ac-Ser-Asp-Glu-	NatA
42	832646	XP_002312268.1	Translation initiation factor eIF-5A	At1g13950	1e^−103^	Cytoplasm	Ac-Ser-Asp-Glu-	NatA
43	835953	XP_002325136.1	Translation initiation factor eIF-5A	At1g13950	1e^−102^	Cytoplasm	Ac-Ser-Asp-Glu-	NatA
44	724093	XP_002315023.1	Translation initiation factor eIF-5A	At1g13950	2e^−101^	Cytoplasm	Ac-Ser-Asp-Glu-	NatA
45	740524	XP_002327184.1	WPP domain-containing protein 2	At1g47200	9e^−40^	Cytoplasm	Ac-Ser-Asp-Ser-	NatA
46	819223	XP_002308228.1	Metallopeptidase M24 protein	At3g51800	0.0	Cytoplasm	Ac-Ser-Ser-Asp-	NatA
47	577003	XP_002322994.1	Metallopeptidase M24 protein	At3g51800	0.0	Cytoplasm	Ac-Ser-Ser-Asp-	NatA
48	836390	XP_002326994.1	T-complex protein 1 alpha subunit	At3g20050	0.0	Cytoplasm	Ac-Ser-Ile-Ala-	NatA
49	819264	XP_002308283.1	Nucleosome assembly protein 1;2	At2g19480	3e^−176^	Cytoplasm	Ac-Ser-Asn-Asp-	NatA
50	736146	XP_002323796.1	Glucose-6-phosphate dehydrogenase	At5g40760	0.0	Cytoplasm	Ac-Gly-Ser-Gly-	NatA
51	641721	XP_002298586.1	Glucose-6-phosphate dehydrogenase	At5g40760	0.0	Cytoplasm	Ac-Gly-Ser-Gly-	NatA
52	825441	XP_002323696.1	Phosphoglycerate mutase	At1g09780	0.0	Cytoplasm	Ac-Gly-Ser-Pro-	NatA
53	739967	XP_002326437.1	26S proteasome AAA-ATPase subunit RPT1a	At1g53750	0.0	Cytoplasm	Ac-Gly-Ser-Gly-	NatA
54	819597	XP_002309623.1	DEAD-box ATP-dependent RNA helicase 56	At5g11200	0.0	Cytoplasm	Ac-Gly-Glu-Thr-	NatA
55	578196	XP_002324856.1	DEAD-box ATP-dependent RNA helicase 56	At5g11200	0.0	Cytoplasm	Ac-Gly-Thr-Asn-	NatA
56	735121	XP_002322743.1	Ser/thr protein phosphatase PP2A catalytic subunit	At3g58500	0.0	Cytoplasm	Ac-Gly-Thr-Asn-	NatA
57	739954	XP_002326738.1	Putative trehalose-6-phosphate synthase	At4g17770	0.0	Cytoplasm	Ac-Val-Ser-Arg-	NatA
58	553698	XP_002303278.1	CASC3/Barentsz eIF4AIII binding protein	At1g80000	1e^−98^	Cytoplasm	Ac-Thr-Lys-Val-	NatA

a NA represents “not available”.

The two enzymes of PDFs and MAPs are involved in NME, whereas Nats are responsible for N^α^-acetylation of proteins in yeast and humans [1], [3]–[5], [18]–[19]. Based on these observations, NME and N^α^-acetylation of poplar may also be dependent on these enzyme orthologs, which are described in the following section.

### Identification of the Enzymes Involved in NME Processing of these Poplar Acetylated Proteins

PDF and MAP activities are successively needed for NME of plastid-encoded proteins [1], [5], [8]. As for nuclear-coded proteins in eukaryotes, NME only needs MAP enzyme activities, which proteolytically remove the N-terminal Met [4], [6]. To determine which enzyme orthologs were involved in NME of these acetylated proteins from poplar, the subcellular location of these corresponding genes of identified acetylated proteins were determined by searching the *Populus trichocarpa* genome database (http://genome.jgi.doe.gov/poplar/). As a result, we found that all identified acetylated proteins were products encoded by nuclear genes. Accordingly, It could be proposed that MAPs, but not PDFs, represent the only enzymes responsible for NME of the 47 identified acetylated proteins from group (ii). However, further efforts are required to be determined which MAPs function as NME of these proteins in poplar.

**Figure 1:**
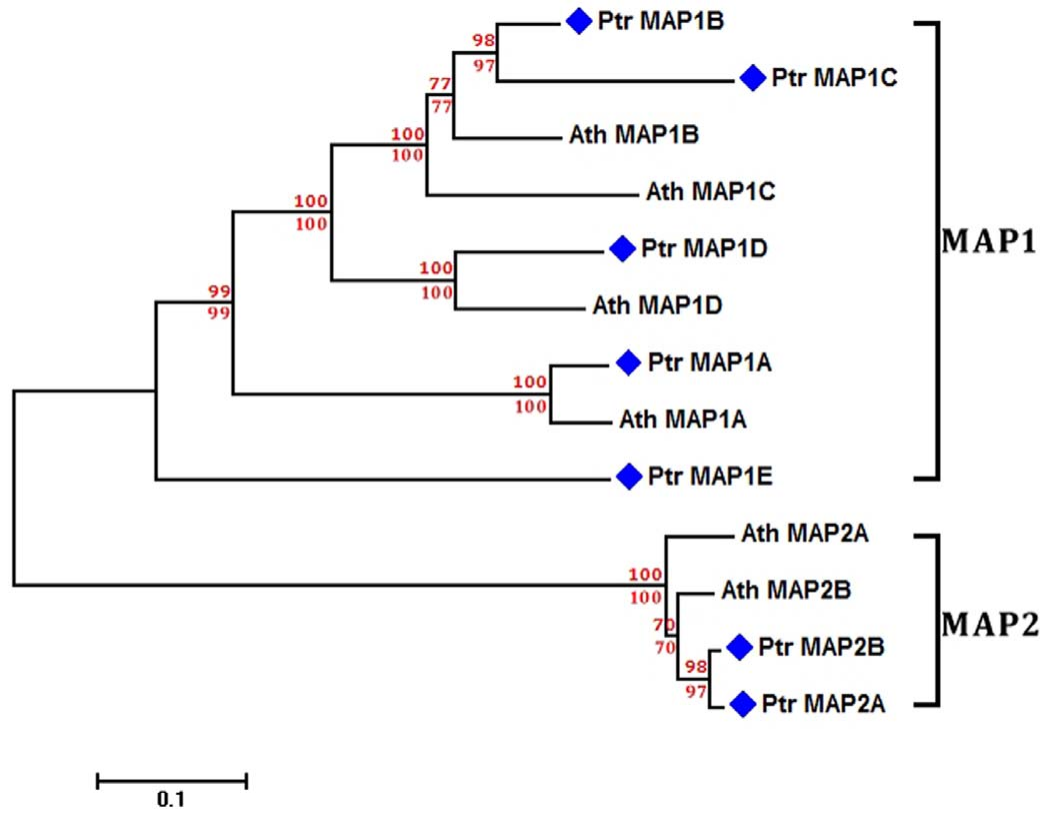
Phylogenetic analysis of MAPs. A phylogenetic tree was generated with the neighbor-joining method using the p-distance in MEGA version 5.04 [47] from all complete MAP protein sequences of *Arabidopsis* and poplar. Branch lengths are proportional to the amino acid distances along each branch. The neighbor-joining bootstrap values for clusters supported above the 50% level are indicated in the red font above the branches, whereas minimum-evolution bootstrap values above the 50% level are presented in red font below the branches. The blue diamonds are highlighted at the front of all MAPs from *Populus*. PtrMAP1A (XP_002320050.1); Ptr MAP1B (XP_002311439.1); Ptr MAP1C (JGI protein ID, 225648); Ptr MAP1D (XP_002306464.1); Ptr MAP1E (XP_002336218.1); Ptr MAP2A (XP_002301676.1); Ptr MAP2B (XP_002305888.1); Ath MAP1A (AAP37849.1); Ath MAP1B (AEE28993.1); Ath MAP1C (AEE77063.1); Ath MAP1D (AEE86740.1); Ath MAP2B (AEE79999.1); Ath MAP2A (AAP21284.1).

Although there has been systematic and comprehensive characterization of MAPs in *Arabidopsis* [4]–[5], until now such information has not been documented in poplar. To clearly obtain all members of the MAP families in *Populus*, the *P. trichocarpa* protein sequence data [20] was exploited as a query file for searching across the Conserved Domain Database (CDD) [21]. We found five non-redundant putative MAP1s that significantly matched the MetAP1 domain (cd01086), whereas two MAP2s were found to significantly match the MetAP2 domain (cd01088) (File S3 and Table 2). A separate phylogenetic tree was generated from all complete MAP protein sequences of *Arabidopsis* and poplar (Figure 1). Phylogenetic analysis demonstrated that two distinct clusters are present, including MAP1 and MAP2 clusters, which are respectively encoded by evolutionarily divergent genes (Figure 1). These identified poplar MAPs were denominated in accordance with their MAP orthologues with the closest evolutionary relatedness in *Arabidopsis*. Consequently, one member of poplar MAP1s (730835) has the closest evolutionary relation with *Arabidopsis* MAP1A (Ath MAP1A, At2g45240), and was therefore termed poplar MAP1A (Ptr MAP1A) (Figure 1 and Table 2). Notably, another member of poplar MAP1s (588331) was considered a novel member of MAP1s because of the divergence between this protein and other MAP1s (MAP1A-D). This MAP1 was termed Ptr MAP1E (Figure 1 and Table 2). Furthermore, we found that the MAP1 domain of Ptr MAP1E has high sequence similarity with other MAP1s of *Arabidopsis* and poplar (Figure S1a), whereas the absence of the N-terminal extension was only present in Ptr MAP1E, which could represent its divergence from MAP1A-D of *Arabidopsis* and poplar (Figure S1a). Surprisingly, Ptr MAP2A and Ptr MAP2B, as well as Ath MAP2A and Ath MAP2B share near-identical amino acid sequences, suggesting a conservation of function (Figure S1b).

**Table 2:** All predicted MAPs present in *Populus trichocarpa* genome.

JGI ACS. number	Protein ID	Domain name in CDD (ID)	The closest relatedness member in *Arabidopsis*	Novel simplified nomenclature	Subcellular location of the protein
730835	XP_002320050.1	MetAP1 (cd01086)	Ath MAP1A (At2g45240)	Ptr MAP1A	Cytoplasm
720677	XP_002311439.1	MetAP1 (cd01086)	Ath MAP1B (At3g25740)	Ptr MAP1B	Mitochondrion
225648	NA^a^	MetAP1 (cd01086)	Ath MAP1C (At1g13270)	Ptr MAP1C	Chloroplast
760457	XP_002306464.1	MetAP1 (cd01086)	Ath MAP1D (At4g37040)	Ptr MAP1D	Mitochondrion
588331	XP_002336218.1	MetAP1 (cd01086)	Ath MAP1A (At2g45240)	Ptr MAP1E	Mitochondrion
552920	XP_002301676.1	MetAP2 (cd01088)	Ath MAP2B (At3g59990)	Ptr MAP2A	Cytoplasm
198596	XP_002305888.1	MetAP2 (cd01088)	Ath MAP2A (At2g44180)	Ptr MAP2B	Cytoplasm

a NA represents “not available”.

**Figure 2:**
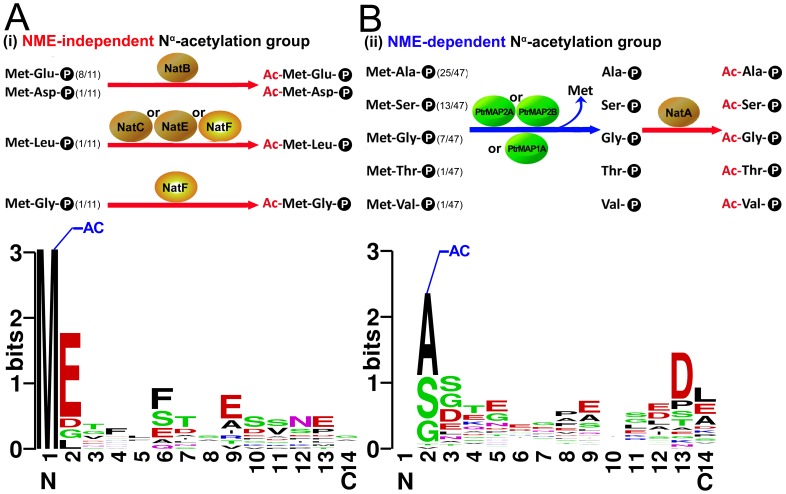
These identified protein acetylation reaction and sequence alignment of the acetylation sites. A, the acetylation reaction and sequence alignment of the 11 N^α^-acetylated proteins that retained their Met residue from group (i). B, the acetylation reaction and sequence alignment of the acetylated sites of the 47 N^α^-acetylation proteins where the Met residue has been removed from group (ii). “-AC” represents N^α^-acetylation, and in B, the blank at position 1 represents the removed of the N-terminal Met residue; sequence alignment of the acetylation sites was made by Weblogo program.

In *Arabidopsis*, three organelle-targeted MAPs (MAP1B, MAP1C and MAP1D), and three cytosolic MAPs (MAP1A, MAP2A and MAP2B) have been characterized as members of the NME machinery [1], [4]–[5]. However, the role of these MAPs in NME of poplar remains unclear. Using TargetP [22], it was predicted that, in poplar, the two Ptr MAP2s (Ptr MAP2A and Ptr MAP2B) are specifically targeted to the cytoplasm, whereas PtrMAP1s is targeted to both the organelles (PtrMAP1B-E) and the cytoplasm (PtrMAP1A). Due to the absence of any plastid-encoded proteins (Table 1), these proteins from the NME-dependent N^α^-acetylation group (ii), should be subjected to NME by the three cytosolic MAPs (PtrMAP1A, PtrMAP2A and PtrMAP2B) in poplar (Figure 2 and Table 2).

### The Nats Involved in N^α^-acetylation of the Identified Poplar Proteins

Confirmation of acetylation sites are recognized footprints of Nat activities. Eukaryotic proteins subject to N^α^-acetylation have a variety of N-terminal sequences and these extracted consensus motifs reflect the activity of particular Nats. In order to identify the Nat orthologs responsible for N^α^-acetylating these poplar proteins, the N-terminal 14 amino acids from the N^α^-acetylated proteins of NME-independent N^α^-acetylation group (i) and NME-dependent N^α^-acetylation group (ii) were respectively aligned in a sequence logo plot using WebLogo [23] (Figure 2A and 2B). From the alignment of 11 acetylated proteins belonging to the group (i), we extracted four motifs, Met-Glu- (8/11), Met-Asp- (1/11), Met-Leu- (1/11), Met-Gly- (1/11) (Figure 2A). The first two striking enrichments of acetylation site motifs (Met-Glu- and Met-Asp-) match the previously identified NatB substrate motifs identified in yeast and humans (Figure 2A). The third enriched motif (Met-Leu-) was consistent with one of the substrate motifs of NatC, NatE or NatF identified in yeast and humans [12] (Figure 2A). The last substrate motif, Met-Gly-, was assigned to one of the NatF substrate motifs of yeast [2], [12], [15], [24] (Figure 2A).

Removal of the N-terminal Met of nuclear-encoded proteins by cytosolic MAP frequently leads to N^α^-acetylation of the resulting N-terminal Ala, Val, Ser, Thr, or Cys residues [9]. We found that similar events were also present for the 47 acetyl-proteins of group (ii), based on the alignment of their N-terminus (Figure 2B). As illustrated in Figure 2B, there was an amino acid preference at positions two and three ([Ala/Ser/Gly/Thr/Val]-[Ser/Gly/Asp/Glu] respectively) with the first position representing the removed Met. Accordingly, the preference likely represents a combination of consensus motifs for the MAPs and Nats. Furthermore, these acetylated residues are represented by five amino acid residues: Ala (25/47), Ser (13/47), Gly (7/47), Thr (1/47) and Vla (1/47), which is consistent with the substrate profiles of NatA in yeast [9], [12], [24] (Figure 2B). This result suggests that N^α^-acetylation of these 47 proteins from group (ii) most likely involves the corresponding NatA orthologs in poplar. In summary, the major acetylases involved in the acetylation of these proteins of poplar are NatA (acetylates 47 (81%) proteins) and NatB (acetylates nine (>15%) proteins) orthologs (Figure 2A and 2B).

### Identification of Nats in Poplar and *Arabidopsis*

Although we suggested that NatA, NatB, NatC, NatE and NatF orthologs may be involved in acetylation of the identified proteins according to recognized substrate motifs by known Nats found in yeast and humans [12], it still remains unexplored whether the poplar genome contains genes encoding similar Nat orthologs to those found in yeast and humans. In order to precisely obtain all members of each type of Nat orthologs in *Populus*, domain files representing subunits of individual types [25] were exploited as queries to identify the Nat orthologs in the *P. trichocarpa* genome [20]. As a result, we identified 16 non-redundant putative Nat orthologous proteins that were composed of all catalytic and auxiliary subunits of the six types of Nats (NatA-F) (Table 3). Except for the NatD catalytic subunit (Ptr Naa40p), N-terminal amino acid sequences of these identified Nat catalytic subunits orthologs showed that these proteins have the consensus acetyl coenzyme A (AcCoA) binding motif, RxxGxG/A, which is a sequence feature of the N-acyltransferase superfamily (Figure S2). To further characterize the observation in other model plants, we extended the search to the *Arabidopsis* protein sequence database (http://www.arabidopsis.org/). Similarly, the *Arabidopsis* genome also contains the genes encoding the six types of Nats (NatA-F) (Table 3). Each Nat catalytic subunit in poplar and *Arabidopsis* shares high sequence similarity to their counterparts in yeast and humans (Figure S3a–f), suggesting that they are highly conserved from lower to higher eukaryotes. Notably, the AcCoA binding motif RxxGxG/A is also present in catalytic subunit of each NatA, NatB, NatC, NatE and NatF, whereas this motif is absent in catalytic subunit of NatD (Naa40p) from *Arabidopsis*, poplar, yeast and human (Figure S3a–f).

**Table 3:** All predicted Nats present in *Arabidopsis* and *Populus* genomes.

Type	Sub -units	Primary	Synonyms	*Arabidopsis*			*Populus*			
				Accession No.	Novel simplified nomenclature	Subcellular Location	JGI protein ID	Refseq protein ID	Novel simplified nomenclature	Subcellular location
NatA	CS^a^	Naa10p Naa11p	Ard1p Ard2p	AT5G13780	Ath Naa10p	Secretory pathway	650021	XP_002314058.1	Ptr Naa10p	Secretory pathway
							641307	XP_002298415.1	Ptr Naa11p	Secretory pathway
	AS^b^	Naa15p Naa16p	Nat1p	AT1G80410	Ath Naa15p	Cytoplasm	548659	XP_002299630.1	Ptr Naa15p	Cytoplasm
							553694	XP_002304180.1	Ptr Naa16p	Cytoplasm
NatB	CS	Naa20p	Nat3p	AT1G03150	Ath Naa20p	Cytoplasm	818659	XP_002307586.1	Ptr Naa20p	Secretory pathway
							643297	XP_002300841.1	Ptr Naa21p	Cytoplasm
	AS	Naa25p	Mdm20p	AT5G58450	Ath Naa25p	Cytoplasm	571859	XP_002319956.1	Ptr Naa25p	Cytoplasm
NatC	CS	Naa30p	Mak3p	AT2G38130	Ath Naa30p	Cytoplasm	727122	XP_002317002.1	Ptr Naa30p	Cytoplasm
							642436	XP_002298931.1	Ptr Naa31p	Cytoplasm
	AS I	Naa35p	Mak10p	AT2G11000	Ath Naa35p	Cytoplasm	560565	XP_002308056.1	Ptr Naa35p	Cytoplasm
	AS II	Naa38p	Mak31p	AT1G65700	Ath Naa38p	Cytoplasm	641478	XP_002299990.1	Ptr Naa38p	Cytoplasm
NatD	CS	Naa40p	Nat4p	AT1G18335	Ath Naa40p	Cytoplasm	729076	XP_002318313.1	Ptr Naa40p	Cytoplasm
NatE	CS	Naa50p	Nat5p	AT5G11340	Ath Naa50p	Cytoplasm	737117	XP_002324274.1	Ptr Naa50p	Cytoplasm
							654093	XP_002308640.1	Ptr Naa51p	Cytoplasm
NatF	CS	Naa60p	Nat15	AT5G16800	Ath Naa60p	Cytoplasm	834607	XP_002319255.1	Ptr Naa60p	Cytoplasm
				AT3G02980	Ath Naa61p	Cytoplasm	665408	XP_002325388.1	Ptr Naa61p	Mitochondria

a CS denotes catalytic subunit of Nat;

b AS represents auxiliary subunit of Nat.

Although we identified the NatD orthologs of *Arabidopsis* (Ath Naa40p) and poplar (Ptr Naa40p), which is homologous to yeast Nat4p (Table 3), NatD activities of poplar were not observed in this study since N^α^-acetylation status of the NatD substrates, histones H2A and H4 [26], has been not determined in the MS experiment. Therefore, further experiments are required to confirm such activity for Naa40p orthologs in poplar. In summary, it has been suggested that poplar and *Arabidopsis* should share the common NatA-F system present in yeast and humans.

## Discussion

The exact NPM including NME and N^α^-acetylation has been well characterized for yeast. This focus on yeast is primarily because of the accessibility of mutants involved in the pathway. In contrast, negligible progress has been made using woody plants, such as poplar. Furthermore, MS for peptide-based proteomics combined with selective enrichment technologies have been widely used for simultaneous identification of acetylated proteins of yeast and humans [27]–[28]. Based on the large amount of data identifying exact acetylation sites, the substrate spectra of enzymes involved in the pathway process have been well characterized, resulting in the promotion of research targeting the entire NPM [19]. In combination, these techniques and methods enable large-scale identification and analysis of acetylated proteins.

It is noteworthy that TiO_2_ column was considered as one of most effective methods for selective enrichment of phosphopeptides based on the strong specific interaction between TiO_2_ and phosphate groups on the molecule of phosphopeptides. For this reason, we had used mass spectrometry combined with TiO_2_ phosphopeptide-enrichment strategies to investigate the phosphoproteome of dormant terminal buds in poplar (*Populus simonii* × *P. nigra*) [29]. As a result, 161 phosphopeptides with 161 unique phosphorylated sites from 151 proteins were identified [29]. Surprisingly, we identified 51 N-terminally acetylated peptides from 58 proteins with high confidence, among which fourteen N^α^-acetylated peptides (27.5%, 14/51) were also occurred on phosphorylation in this study (Table 1 and File S2). To explore and clarify why these N-terminal acetylated peptides and phosphopeptides were together enriched using this approach of TiO_2_ affinity, we made *in silico* analysis of the theoretical pI and acidic amino acid composition (including D and E) for these N^α^-acetylated peptides without the occurrence of phosphorylation events using ProtParam tool (http://web.expasy.org/protparam/). We do so mainly because highly acidic peptides or those containing multiple acidic residues tend to absorb with TiO_2_ despite the presence of a number of improved TiO_2_ phosphopeptide-enrichment procedures [30]–[32]. It is found that almost all of the N^α^-acetylated peptides could be considered as highly acidic peptides or pepides containing multiple acidic residues because of their low theoretical pI or high acidic amino acid composition (File S5). Accordingly, we thought that the enrichment of these N^α^-acetylated peptides using TiO_2_ microcolumn should mainly be due to the strong specific interaction between their additional phosphate groups or multiple acidic residues and TiO_2_. However, it should be very interesting that further experiment are now required to investigate whether interaction between the N-acetyl moiety of protein and TiO_2_ was occurred and functioned on this process of enrichment.

Compared with phosphopeptide-enrichment strategies, to date there are still no more suitable methods applied in the N-terminal acetylpeptide-enrichment strategies. Such case directly lead to protein N^α^-acetylation remaining poorly explored [33]. To address this, the recent emergence of N-terminomics technologies that allow isolation of protein N-terminal peptides, have greatly boosted the field of N^α^-acetylation [33]. However, these technologies might go through many cumbersome and complicated steps, such as N-terminus of the tryptic cleavage products require extensive chemical modification (trinitrobenzene or biotinylation) and/or consecutively repeated separation of the sample [33]–[35]. Therefore, until recently, few data resource about protein N^α^-acetylation could be provided, especially for woody plants. Although 51 unique acetyl-peptides from 58 proteins were accidentally identified in our study of poplar phosphoproteome since phosphate groups or multiple acidic residues within these acetyl-peptides contributed to their affinity with TiO_2_ microcolumn, the site-specific acetylation data could also provide a wealth of valuable resources to assist us decoding NPM mechanisms present in poplar.

### Removal of the N-terminal Met in Poplar Follows the NME Rule

In this study, we used a proteomics approach to investigate the acetylation status of poplar proteins. Fifty-eight N^α^-acetylated proteins were identified and the majority of these proteins (47, >81%) undergo N-terminal Met cleavage and subsequent acetylation of the exposed N-terminal residue at position two. Moreover, the residues (Ala, Ser, Gly, Thr and Vla) at position two comply with the above-mentioned rule of NME. Surprisingly, of the 11 proteins belonging to group (i), we found that the N-terminal Met residue of one PPO (794816) was acetylated (Table 1); however, the adjacent residue at position two was a Gly and facilitated NME (File S2). Specifically, the Met of the N-terminal motif (Met-Gly-) of the poplar PPO should have been removed according to the NME rule; however, it was retained and acetylated. This observation is in accord with a recent study that showed that the Met-Gly- sequence represents a substrate motif of a newly identified NatF-type in yeast [12] (Figure 2A).

### One Extended Nat Catalytic Subunit System Occurs in the Poplar Genome

Currently, six types of Nats (NatA-NatF) represent the full set of enzymes of the Nats system from yeast to humans [12]. In this study, we found that both *Arabidopsis* and poplar genomes contain the full Nat system composed of NatA-F (Table 3). Most of the Nat catalytic subunits in poplar exist as two paralogous isoforms, such as the NatA catalytic subunits of Ptr Naa10p and Ptr Naa11p, and the NatB catalytic subunits of Ptr Naa20p and Ptr Naa21p. In contrast, only NatD exists as a single protein, Ptr Naa40p (Table 3). Conversely, no single Nat catalytic subunit of yeast contains paralogous isoforms, only one NatA catalytic subunit in humans contains paralogous isoforms (i.e., Naa10p and Naa11p) and one NatF catalytic subunit of *Arabidopsis* contains paralogous isoforms (Ath Naa60p and Ath Naa61p) (Table 3 and File S4). This observation suggests that the genes encoding Nat catalytic subunits in poplar have expanded. This expansion, often present on a large number of *Populus* multi-gene families, could have occurred from multiple gene duplication events, including segmental duplication and tandem duplication events [20]. The presence of more Nat subunit genes in the *Populus* genome may reflect a greater requirement for acetylation of proteins. A detailed schematic view of the number of paralogous isoforms of each Nat catalytic subunit from the four organisms is provided in Figure S4.

### Cytosolic Nat Isoforms Present in Poplar

Following Met cleavage by MAP, the exposed small side-chain amino acid, Ser-, Ala-, Thr-, Val-, Gly- or Cys-, is often further acetylated by NatA, a Nat enzyme present in either the cytoplasm [3], [19], [36] or the chloroplast of *Arabidopsis* [6], [37]–[38]. These data indicate that there should be both chloroplastic and cytosolic isoforms of NatA present in *Arabidopsis*. However, we have identified only one *Arabidopsis* NatA complex consisting of one catalytic subunit (AT5G13780, Ath Naa10p) and one auxiliary subunit (AT1G80410, Ath Naa15p) (Table 3). Surprisingly, TargetP prediction indicates that Ath Naa10p is secreted, whereas Ath Naa15p is targeted to the cytoplasm in *Arabidopsis* [18], [22] (Table 3). Furthermore, cytosolic isoforms of NatA with Nat activity are composed of both Ath Naa10p and Ath Naa15p, and the chloroplastic isoforms of NatA only consist of Ath Naa10p [18], [22]. Similarly, the secreted catalytic subunits (650021, Ptr Naa10p and 641307, Ptr Naa11p) and cytosolic auxiliary subunits (548659, Ptr Naa15p and 553694, Ptr Naa16p) are also present in poplar (Table 3). Thus, we propose that the single presence of one of the two catalytic subunits “Ptr Naa10p and Ptr Naa11p” combination with auxiliary subunits of Ptr Naa15p and Ptr Naa16p should be the cytosolic isoform forms of NatA in poplar.

According to the analysis of the substrates profile, NatA should be major N-terminal Nats, which could be responsible for acetylating 81% of the identified proteins (Figure 2B and Table 1). Identifying which NatA isoforms carry out the acetylation of these proteins are important. To address this, information about subcellular location of the identified poplar proteins was obtained using TargetP [22], and by a comparison of their best hits in *Arabidopsis* with the latest plant plastid database (PPDB) [18]. As a result, among the 47 acetylated proteins from group (ii) by NatA, all proteins were targeted to cytoplasm, while no any proteins targeted to the chloroplast were found (Table 1), suggesting that these acetylation events should be carried out by cytosolic NatA isoform and not by chloroplastic NatA isoform. Chloroplastic and cytosolic isoforms of NatB had respectively been found in *Chlamydomonas reinhardtii* [39] and *Arabidopsis* [18]. However, it is noteworthy that the two catalytic subunits of NatB in poplar, one is secreted catalytic subunit (Ptr Naa20p) and other one is cytosolic catalytic subunit (Ptr Naa21p), whereas the only one auxiliary subunit of NatB (Ptr Naa25p) in poplar is targeted to cytoplasm (Table 3). Similar to NatA, NatB of poplar should also exist in the forms of either chloroplastic or cytosolic isoforms, where the individual Ptr Naa20p or Ptr Naa21p combination with auxiliary subunit (Ptr Naa25p) could compose cytosolic isoform of poplar NatB.

In summary, we confirm that the N-terminal Met residues of these proteins from the NME-independent N^α^-acetylation group (i), should be directly N^α^-acetylated by cytosolic NatB, NatC, NatE and cytosolic NatF. And that the second N-terminal residue of these proteins from the NME-dependent N^α^-acetylation group (ii), should be N^α^-acetylated by cytosolic NatA following the subjected to NME by three cytosolic MAPs (PtrMAP1A, PtrMAP2A and PtrMAP2B) in poplar (Figure 2A and 2B).

### The Biological Significance of Nα-acetylation of these Proteins during the Dormancy of Poplar

For many years, it was thought that N^α^-acetylation protected proteins from degradation [40]–[41]. On the contrary, N-terminal acetylated Met residues were recently found to be involved in creating degradation signals: a ubiquitin ligase, Doa10, recognizes N^α^-acetylated proteins and ubiquitinates the protein, thereby marking it for degradation [9]. Although these two hypotheses predict opposite functional outcomes for N^α^-acetylation and thus appear to be contradictory, both mechanisms may take place side-by-side in the cell, each functioning to a specific subsets of proteins under defined conditions [42]. Accordingly, it was proposed that the functional consequences of N^α^-acetylation of these identified proteins during dormancy of poplar may be dependent on each specific protein and its cellular state. However, the current major challenge is to determine the specific functions of each individual acetylated protein during the dormancy of poplar.

In conclusion, we have identified 58 N^α^-acetylated proteins using a tandem MS method combined with TiO_2_ acetylpeptide-enrichment strategies. Based on the analysis of the N-terminus of these proteins, we confirm that poplar possesses the analogous NPMs including NME rule and Nat system to other eukaryotes. Furthermore, we also confirm that the acetylation reactions and their involving enzymes of these identified proteins in poplar. Further experiments are now required to confirm that these specific MAP and Nat enzymes interact with the identified acetylation proteins *in vivo*. A promising way forward is to widely identify and characterize the dynamics of protein acetylation in response to environmental changes, applying specialized targeted quantitative acetylation proteomics tools.

## Materials and Methods

### Chemicals and Reagents

HPLC-grade acetonitrile (ACN) was purchased from JTBaker (Thomas Scientific, Swedesboro, NJ, USA). HPLC-grade water was produced with a Milli-Q A10 system from Millipore (Billerica, MA, USA). Iodoacetamide (IAA) and dithiothreitol (DTT) were obtained from Acros Organics (Morris Plains, NJ, USA). ModiWed sequencing-grade trypsin was supplied by Promega (Madison, WI, USA). Pharmalyte, protease-inhibitor cocktail and the 2-D Quant kit were obtained from Amersham Pharmacia Biotech (Uppsala, Sweden). All other reagents were purchased from Sigma (St Louis, MO, USA).

### Plant Material

Dormant terminal buds were harvested from *Populus simonii* × *Populus nigra* trees in Harbin**,** China, (E126°37′ N45°42′) at the end of December 2009. The samples were frozen in liquid nitrogen and stored at −80°C until protein extraction was required.

### Preparation of Total Protein

The dormant terminal buds were homogenized into a fine powder in liquid nitrogen and resuspended at −20°C with 10% (w/v) trichloroacetic acid (TCA) in cold acetone containing 0.07% (v/v) 2-mercaptoethanol for a minimum of 2 h. The mixture was centrifuged at 40,000 *g* at 4°C for 1 h and the precipitates were washed with cold acetone containing 0.07% (v/v) 2-mercaptoethanol. The pellets were dried by vacuum centrifugation and dissolved in 7 M urea, 2 M thiourea, 20 mM dithiothreitol, 1% (v/v) protease-inhibitor cocktail, 0.2 mM Na_2_VO_3_ and 1 mM NaF at room temperature for 2 h before centrifugation at 40,000 *g* at 4°C for 1 h. The resulting supernatant was collected and stored at −80°C until further use. The total protein content of the samples was quantified using a 2-D Quant kit.

### In-solution Protein Digestion

Total proteins were digested as previously described [43]–[44]. Briefly, after adjusting the pH of the total protein solution to pH 8.5 with 1 M ammonium bicarbonate, the sample was reduced for 45 min at 55°C by adding DTT to a final concentration of 10 mM, and then carboxyamidomethylated in 55 mM iodoacetamide for 30 min at room temperature in the dark. CaCl_2_ was then added to a final concentration of 20 mM. Endoprotease Lys-C was added to a final substrate-to-enzyme ratio of 100∶1 and the reaction was incubated at 37°C for 12 h. The Lys-C digestion was added to 1 M urea with 100 mM ammonium bicarbonate and modified trypsin was added to a final substrate-to-enzyme ratio of 50∶1. The trypsin digestion was incubated at 37°C for 12 h. After digestion, the peptide mixture was acidified with formic acid for further MS analysis. Samples that were not immediately analyzed were stored at −80°C.

### Enrichment of Acetylated Peptides Using a TiO_2_ Microcolumn

The TiO_2_ microcolumns were packed as previously described [30]. A small plug of C_8_ material was stamped out of a 3 M Empore C_8_ extraction disk using an HPLC syringe needle and placed at the small end of the GELoader tip. The C_8_ disk served only as a frit to retain the TiO_2_ beads within the GELoader tip. The TiO_2_ beads were suspended in 100% ACN and an aliquot of this suspension (depending on the size of the column) was loaded onto the GELoader tip. Gentle air pressure created by a plastic syringe was used to pack the column. The TiO_2_ microcolumn was equilibrated with loading buffer (40 µl; 80% ACN/5% TFA/saturated phthalic acid solution) and the trypsin-digested peptide mixture diluted with loading buffer was then loaded onto the column. The TiO_2_ microcolumn was washed once with loading buffer (40 µl) and three times with washing solution (40 µl; 80% ACN/2% TFA). The solvent used for washing and loading the sample onto the TiO_2_ microcolumn contained organic solvent (80% ACN), which abrogates the adsorption of peptides to the C_8_ material [45]. The bound peptides were eluted twice with 40 µl of ammonium bicarbonate, pH >10.5, and then with 10 µl of 30% ACN. The eluted peptides were lyophilized and dissolved in 1% formic acid before MS analysis.

### NanoUPLC-ESI-MS/MS

NanoUPLC-ESI-MS/MS was performed with a splitless nanoUPLC (10 kpsi nanoAcquity; Waters) coupled to a Synapt high-resolution mass spectrometer with a nanospray ion source (Waters). The program MassLynx (version 4.1; Waters) was used for data acquisition and instrument control. A symmetric C_18_ 5-µm, 180-µm × 20-mm pre-column and a BEH C_18_ 1.7-µm, 75-µm × 250-mm analytical reversed-phase column (Waters) were used. The mobile phases were (A) 100% H_2_O/0.1% formic acid and (B) 100% ACN/0.1% formic acid. The samples were initially transferred in an aqueous 0.1% formic acid solution to the pre-column with a flow rate of 5 µl/min for 3 min. The peptides were separated by a gradient of 5?40% mobile phase B over 90 min at a flow rate of 200 nl/min, followed by a 10-min rinse with 90% mobile phase B. The column was re-equilibrated using the initial conditions for 20 min. The lock mass was delivered from the auxiliary pump of the NanoAcquity pump with a constant flow rate of 400 nl/min at a concentration of 100 fmol/µl of (Glu1) fibrinopeptide B to the reference sprayer of the NanoLockSpray source of the mass spectrometer. All samples were analyzed in triplicate. Data-dependent acquisition was performed in the positive ion mode. MS spectra were acquired for 1 s from mass-to-charge ratios (*m/z*) of 350 to 1990. Two of the most intense precursor ions that were doubly or triply charged were selected from *m/z* 350 to 1990. MS/MS spectra generated with collision-induced dissociation were acquired for 2 s from *m/z* 50 to 1990. The collision energy was automatically calculated based on the peptide charge and *m/z*; a dynamic exclusion window was applied that prevented the same *m/z* from being selected for 2 min after its acquisition. The mass tolerance in the MS and MS/MS modes was 15 and 50 ppm, respectively. The candidate acetylpeptides were initially assigned by ESI-MS/MS using 42-Da mass increments per acetyl moiety relative to the unmodified peptides.

### Data Analysis and Mascot Database Search

The MS/MS data were converted to a pkl file format using the ProteinLynx software (Waters) and the resulting pkl file was searched against the JGI *Populus trichocarpa* v1.1 (http://genome.jgi-psf.org/Poptr1_1/Poptr1_1.home.html) protein sequence database using an in-house Mascot server (version 1.8). Two missed cleavage sites were allowed: acetylation, carbamidomethylation, methionine oxidation and phosphorylation of serine/threonine/tyrosine of the N-terminus of the protein were accepted as variable modifications. The FDR is 0.00% for peptide matches above the identity threshold and 0.36?0.85% for peptide matches above the homology or identity threshold.

### Bioinformatics

The complete protein sequence database of poplar was downloaded from *Populus trichocarpa* v1.1 (www.jgi.doe.gov/poplar). Using a custom Perl program, all the acetylated protein sequences were extracted from the protein databases by their protein ID identifiers. These protein sequences with the conserved domains of MetAP1 (cd01086) and MetAP2 (cd01088) were respectively considered as the family members of MAP1s and MAP2s by searching the *P. trichocarpa* protein sequence data [20] across the Conserved Domain Database (CDD) [21].

Each subunit of NatA-F orthologs in *Populus* was identified according to the following contents. First, Hidden Markov Model (HMM) profile files of two subunits Mdm20 (PF09797) and Mak10 (PF04112) were obtained from the Pfam database (http://pfam.sanger.ac.uk/). Second, these known protein sequences representing each subunits from the other nine Nat orthologs subunits of various organisms were respectively extracted from the UniProt database (http://www.uniprot.org), and then aligned using the ClustalW program [46]. Subsequently, their HMM profile files were respectively in-house established using the hmmbuild command of the HMMER (v 3.0) software [25]. Finally, HMM profile files of each Nat orthologs subunits were searched against the poplar protein database [20] using the hmmer search command of the HMMER (v 3.0) software [25].

Multiple sequence alignments of the full-length protein sequences were performed using the ClustalW program in the BioEdit software with default parameters. Based on these aligned sequences, the unrooted phylogenetic trees were constructed using the MEGA 5.0 software [47] and the Minimum Evolution method with the parameters p-distance and completed deletion. The reliability of the phylogenetic tree was estimated using a bootstrap value with 1000 replicates.

All non-redundant (Nr) best hits in *Arabidopsis* with the lowest expected values were collected by searching for these identified poplar protein sequences across the REFseq Nr *Arabidopsis* protein database using the BLASTP program of NCBI (http://www.ncbi.nlm.nih.gov/). These N-terminal acetylated peptides from 58 poplar proteins were aligned at these acetylated amino acid residues, and fourteen positions downstream of the acetylated site were included. The alignment of all the acetylated sites completed by the Weblogo program [23], which is a web-based application designed to generate sequence logos.

## Supporting Information

Figure S1 **Alignment of the amino acid sequence of MAPs from** ***Arabidopsis*** **and poplar.** Color shading represents 70% identical residues among the sequences. Gaps were introduced to ensure maximum identity. **a** amino acid sequence alignment of Ptr MAP1E with MAP1A-D of *Arabidopsis* and poplar. Sequence conservation is highest in the region of the MetAP1 domain (*unmarked*), and these MAP1s had various N-terminal extension sequences (*gray box above sequence alignment*). In particular, the N-terminal extension is absent in Ptr MAP1E. **b** amino acid sequence alignment of MAP2s from poplar and *Arabidopsis*. MAP2s from poplar and *Arabidopsis* share near-identical amino acid sequences, indicating that these MAP2s have a conserved function. The identifiers of the proteins are shown in Table 2.
(JPG)

Figure S2 **Amino acid sequence alignment of all predicted Nat catalytic subunits from poplar.** The consensus acetyl coenzyme A (AcCoA) binding motif sequence RxxGxG/A, where x can be any amino acids, is boxed (red). The identifiers of the proteins are shown in Table 3.
(JPG)

Figure S3 **Amino acid sequence alignments of each Nat catalytic subunit from several eukaryotes.** The consensus acetyl coenzyme A (AcCoA) binding motif sequence RxxGxG/A, where x can be any amino acid, is indicated within the red boxes. Gaps were introduced to ensure maximum identity. Color shading represents 70% identical residues among the sequences. **a** amino acid sequence alignment of the NatA catalytic subunits from poplar, *Arabidopsis*, human and yeast. **b** amino acid sequence alignment of the NatB catalytic subunits from poplar, *Arabidopsis*, human and yeast. **c** amino acid sequence alignment of the NatC catalytic subunits from poplar, *Arabidopsis*, human and yeast. **d** amino acid sequence alignment of the NatD catalytic subunits from poplar, *Arabidopsis*, human and yeast. **e** amino acid sequence alignment of the NatE catalytic subunits from poplar, *Arabidopsis*, human and yeast. **f** amino acid sequence alignment of the NatF catalytic subunits from poplar, *Arabidopsis* and human. The identifiers of the proteins are shown in the Supplemental Table 3 and Table 3.
(JPG)

Figure S4 **Schematic view of the number of paralogous isoforms of each Nat catalytic subunit from the four organisms.**
(JPG)

File S1 **A file that contains all the original MS/MS spectra of acetylpeptides identified in this study.**
(TAR)

File S2 **The detailed information for these identified N^α^-acetylated peptides in poplar.**
(XLS)

File S3 **Predicted poplar MAPs containing MetAP1 and MetAP2 domains.**
(DOC)

File S4 **All previously identified Nats present in yeast and human.**
(DOC)

File S5 (XLS)
