# Supplementary figures and images for: Identification and Analysis of the Acetylated Status of Poplar Proteins Reveals Analogous N-Terminal Protein Processing Mechanisms with Other Eukaryotes
Source: PLoS One. 2013 Mar 11;8(3):e58681. doi: 10.1371/journal.pone.0058681 (PMC3594182; doi:10.1371/journal.pone.0058681)

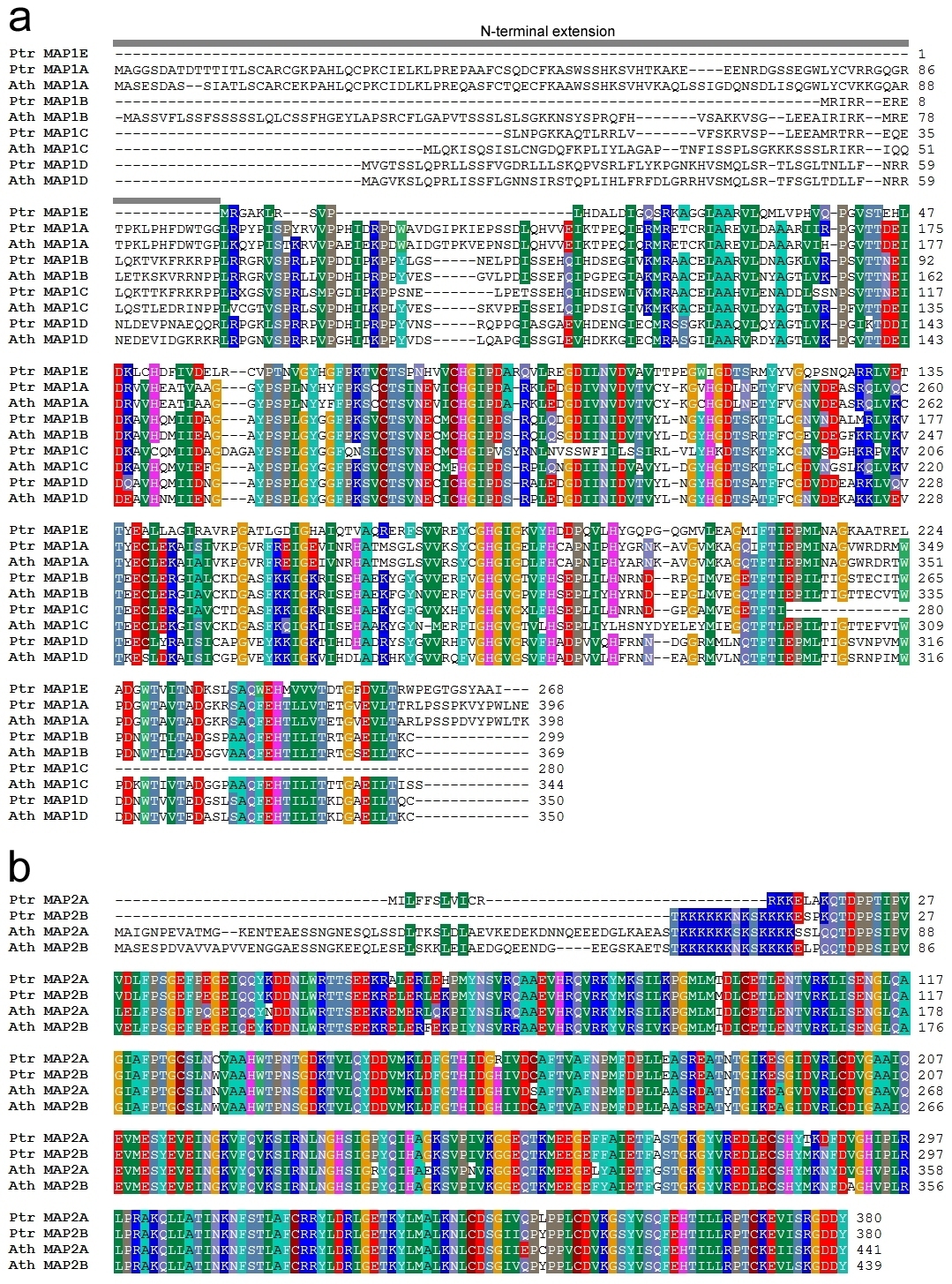

Supplement: Figure S1 — Alignment of the amino acid sequence of MAPs from Arabidopsis and poplar. Color shading represents 70% identical residues among the sequences. Gaps were introduced to ensure maximum identity. a amino acid sequence alignment of Ptr MAP1E with MAP1A-D of Arabidopsis and poplar. Sequence conservation is highest in the region of the MetAP1 domain (unmarked), and these MAP1s had various N-terminal extension sequences (gray box above sequence alignment). In particular, the N-terminal extension is absent in Ptr MAP1E. b amino acid sequence alignment of MAP2s from poplar and Arabidopsis. MAP2s from poplar and Arabidopsis share near-identical amino acid sequences, indicating that these MAP2s have a conserved function. The identifiers of the proteins are shown in Table 2. (JPG) [file pone.0058681.s001.jpg]

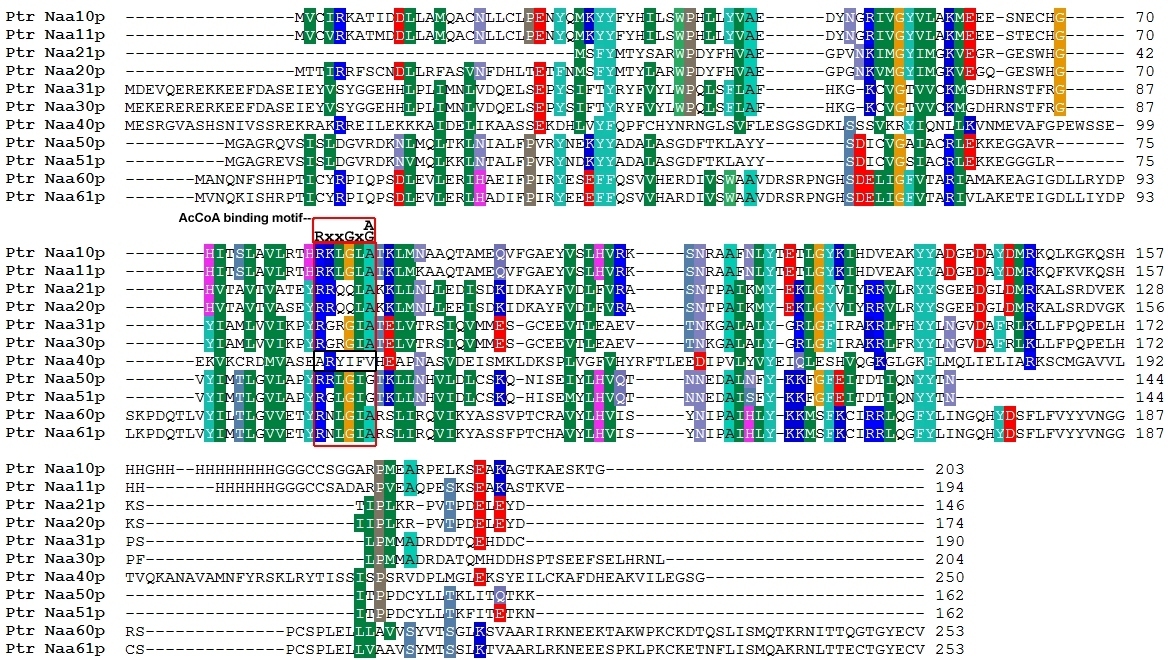

Supplement: Figure S2 — Amino acid sequence alignment of all predicted Nat catalytic subunits from poplar. The consensus acetyl coenzyme A (AcCoA) binding motif sequence RxxGxG/A, where x can be any amino acids, is boxed (red). The identifiers of the proteins are shown in Table 3. (JPG) [file pone.0058681.s002.jpg]

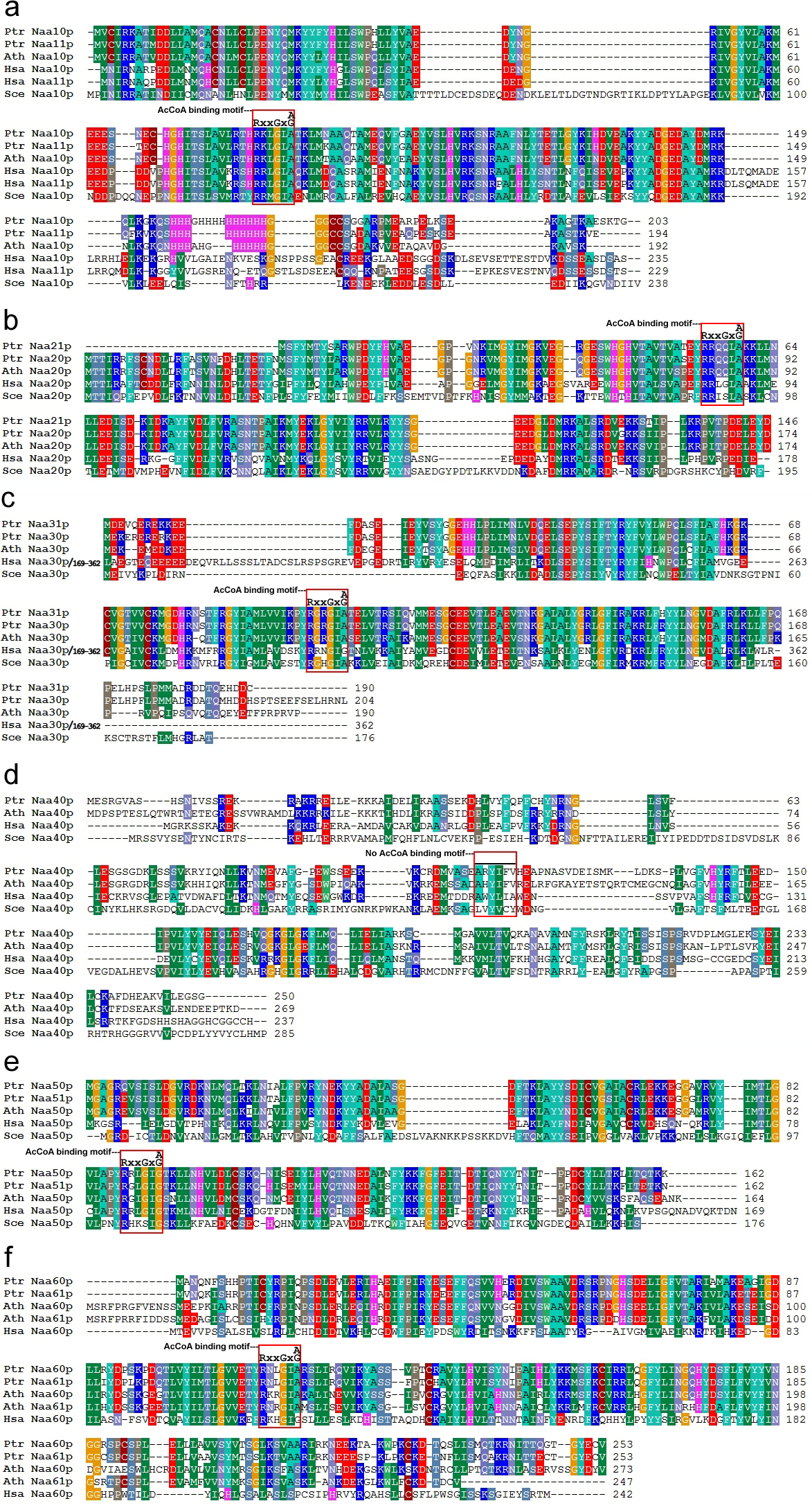

Supplement: Figure S3 — Amino acid sequence alignments of each Nat catalytic subunit from several eukaryotes. The consensus acetyl coenzyme A (AcCoA) binding motif sequence RxxGxG/A, where x can be any amino acid, is indicated within the red boxes. Gaps were introduced to ensure maximum identity. Color shading represents 70% identical residues among the sequences. a amino acid sequence alignment of the NatA catalytic subunits from poplar, Arabidopsis, human and yeast. b amino acid sequence alignment of the NatB catalytic subunits from poplar, Arabidopsis, human and yeast. c amino acid sequence alignment of the NatC catalytic subunits from poplar, Arabidopsis, human and yeast. d amino acid sequence alignment of the NatD catalytic subunits from poplar, Arabidopsis, human and yeast. e amino acid sequence alignment of the NatE catalytic subunits from poplar, Arabidopsis, human and yeast. f amino acid sequence alignment of the NatF catalytic subunits from poplar, Arabidopsis and human. The identifiers of the proteins are shown in the Supplemental Table 3 and Table 3. (JPG) [file pone.0058681.s003.jpg]

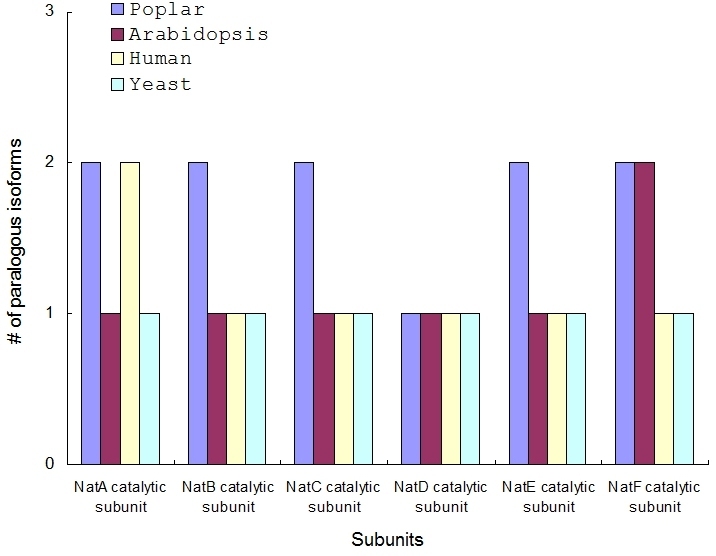

Supplement: Figure S4 — Schematic view of the number of paralogous isoforms of each Nat catalytic subunit from the four organisms. (JPG) [file pone.0058681.s004.jpg]
