# Supplementary material for: Identification and Analysis of the Acetylated Status of Poplar Proteins Reveals Analogous N-Terminal Protein Processing Mechanisms with Other Eukaryotes
Source: PLoS One. 2013 Mar 11;8(3):e58681. doi: 10.1371/journal.pone.0058681 (PMC3594182; doi:10.1371/journal.pone.0058681)
Supplement: File S3 — Predicted poplar MAPs containing MetAP1 and MetAP2 domains. (DOC) [file pone.0058681.s007.doc]

| **File S3** Predicted poplar MetAPs containing MetAP1 and MetAP2 domains | | | | | | | | | |
| --- | --- | --- | --- | --- | --- | --- | --- | --- | --- |
| Query | Hit type | PSSM-ID | From | To | E-Value | Bitscore | Accession | Short name | Superfamily |
| 730835 | specific | 29971 | 145 | 366 | 2.9686E-118 | 342.078 | cd01086 | MetAP1 | cl00279 |
| 760457 | specific | 29971 | 113 | 349 | 8.1335E-113 | 326.67 | cd01086 | MetAP1 | cl00279 |
| 720677 | specific | 29971 | 62 | 298 | 1.1168E-121 | 347.086 | cd01086 | MetAP1 | cl00279 |
| 588331 | specific | 29971 | 17 | 257 | 1.3237E-115 | 330.137 | cd01086 | MetAP1 | cl00279 |
| 225648 | specific | 29971 | 86 | 280 | 1.17278E-48 | 160.264 | cd01086 | MetAP1 | cl00279 |
| 552920 | specific | 29973 | 68 | 376 | 7.7151E-167 | 466.604 | cd01088 | MetAP2 | cl00279 |
| 198596 | specific | 29973 | 68 | 376 | 4.6158E-170 | 475.079 | cd01088 | MetAP2 | cl00279 |
