# Supplementary material for: Identification and Analysis of the Acetylated Status of Poplar Proteins Reveals Analogous N-Terminal Protein Processing Mechanisms with Other Eukaryotes
Source: PLoS One. 2013 Mar 11;8(3):e58681. doi: 10.1371/journal.pone.0058681 (PMC3594182; doi:10.1371/journal.pone.0058681)
Supplement: File S4 — All previously identified Nats present in yeast and human. (DOC) [file pone.0058681.s008.doc]

| **File S4** All previously identified Nats present in yeast and human | | | | | | | |
| --- | --- | --- | --- | --- | --- | --- | --- |
| Type | Subunits | Primary | Synonyms | Yeast (*Saccharomyces cerevisiae*) | | Human (*Homo sapien*s) | |
|  |  |  |  | Accession No. | Novel Simplified Nomenclature | Accession No. | Novel Simplified Nomenclature |
| NatA | CS | Naa10p  Naa11p | Ard1p  Ard2p | P07347 | Sce Naa10p | P41227 | Hsa Naa10p |
|  |  |  |  |  |  | Q9BSU3 | Hsa Naa11p |
|  | AS | Naa15p  Naa16p | Nat1p | P12945 | Sce Naa15p | Q9BXJ9 | Hsa Naa15p |
|  |  |  |  |  |  | Q6N069 | Hsa Naa16p |
| NatB | CS | Naa20p | Nat3p | Q06504 | Sce Naa20p | P61599 | Hsa Naa20p |
|  | AS | Naa25p | Mdm20p | Q12387 | Sce Naa25p | Q14CX7 | Hsa Naa25p |
| NatC | CS | Naa30p | Mak3p | Q03503 | Sce Naa30p | Q147X3 | Hsa Naa30p |
|  | AS I | Naa35p | Mak10p | Q02197 | Sce Naa35p | Q5VZE5 | Hsa Naa35p |
|  | AS II | Naa38p | Mak31p | P23059 | Sce Naa38p | Q95777 | Hsa Naa38p |
| NatD | CS | Naa40p | Nat4p | Q04751 | Sce Naa40p | Q86UY6 | Hsa Naa40p |
| NatE | CS | Naa50p | Nat5p | Q08659 | Sce Naa50p | Q9GZZ1 | Hsa Naa50p |
| NatF | CS | Naa60p | Nat15 | NA | NA | Q9H7X0 | Hsa Naa60p |
| NA represents “not available”; CS denotes catalytic subunit of Nat; and AS represents auxiliary subunit of Nat. | | | | | | | |
